# Red Cell Distribution Width as a Novel Marker for Different Types of Atrial Fibrillation in Low and High Altitude

**DOI:** 10.1155/2019/6291964

**Published:** 2019-03-07

**Authors:** Kaiyue Han, Xiaoling Su, Jiang Liu, Fengcai Yao, FeiYan Lu

**Affiliations:** ^1^Graduate School, Qinghai University, Xining, Qinghai, China; ^2^Department of Cardiology, Qinghai Provincial People's Hospital, Xining, China; ^3^Department of Cardiology, The First Affiliated Hospital of Nanjing Medical University, Nanjing, Jiangsu, China

## Abstract

**Background:**

Increased red cell distribution width (RDW) can predict the incidence and mortality of cardiovascular diseases. However, there are limited data on the relationship between RDW and altitude and the subtype of atrial fibrillation (AF). We investigated the effects of altitude on RDW in patients with different types of AF.

**Methods:**

A total of 303 patients with nonvalvular AF were included. Of these, 156 lived in low altitude (77 paroxysmal AF, PAF; 79 persistent AF, PeAF) and 147 in high altitude (77 paroxysmal AF, PAF; 70 persistent AF, PeAF). In these groups, baseline characteristics, complete blood counts, serum biochemistry, and echocardiography were evaluated. Multivariate logistic regression analysis was conducted to determine the independent predictors of AF at the different altitudes.

**Results:**

In both low and high altitudes, RDW and left atrial diameter (LAD) were higher in AF than control subjects (*P* < 0.05) and higher in persistent AF than paroxysmal AF (*P* < 0.05). Compared with any groups (PAF group, PeAF group, or control group) of low-altitude, RDW and LAD were found higher in high-altitude corresponding groups. Multivariate logistic regression analysis demonstrated that RDW, mean corpuscular volume (MCV), and LAD levels independently associated with AF patients in low altitude (RDW, OR 1.687, 95% CI 1.021–2.789; *P* < 0.05), while in high altitude, RDW, MCV, creatinine (Cr), and LAD were independent predictors for AF patients (RDW, OR 1.755, 95% CI 1.179–2.613; *P* < 0.05).

**Conclusion:**

Elevated RDW levels may be an independent risk marker for nonvalvular AF, affected by type of AF and altitude.

## 1. Introduction

Red blood cell distribution width (RDW) is a parameter of anisocytosis or heterogeneity in the volume of circulating erythrocytes and is traditionally used in laboratory hematology for differential diagnosis of anemias, which is easily available from a standard complete blood cell count (CBC) [1, 2]. Some recent studies have shown that higher RDW level can predict morbidity and mortality of cardiovascular disease, for example, myocardial infarction, heart failure, and atrial fibrillation [3–6]. Atrial fibrillation (AF) is one of the most common arrhythmias worldwide, seriously threatens people's quality of life, and increases the risk of stroke, heart failure, and death. The incidence and prevalence of AF is significantly increased in China [7, 8]. The specific mechanism between the elevated RDW and AF is unclear. Recent studies indicate that inflammatory reaction and oxidative stress play an important role in the connection of RDW and AF [9–11].

High-altitude exposure is known for its strong ultraviolet light and low oxygen pressure. Among them, hypoxia is closely related to the occurrence and development of cardiovascular diseases, mainly manifested by changes in the structure and function of the cardiovascular system, thereby aggravating the occurrence of cardiovascular diseases [12–14]. Some studies shown that the mechanisms of cardiovascular disease associated with altitude include mainly sympathetic activation, inflammatory reaction, and oxidative stress [15–17].

However, there were no data on the association of RDW with different types of nonvalvular AF at different altitude areas. We aimed to investigate the role of RDW as a novel marker for different types of AF at different altitude in China.

## 2. Methods

### 2.1. Study Population

Our study included 303 patients with nonvalvular AF in total, 156 in low altitude (77 paroxysmal AF, PAF; 79 persistent AF, PeAF), 147 in high altitude (77 PAF; 70 PeAF), respectively, and 167 patients without AF (72 in low altitude, 95 in high altitude) matched for sex, age, atherosclerotic risk factors, and history of medicine who were admitted in cardiology department from March 2016 to March 2018 in Second Hospital of Tianjin Medical University and Qinghai Provincial People's Hospital from two cities (Tianjin and Xining), respectively. Tianjin is located at 3.5 m. Xining is located at 2260 m above the sea level. AF was defined as absence of *P* waves and irregular R-R interval in a 12-lead electrocardiogram (ECG) or 24 h Holter recording. The different types of AF were defined according to the ESC guidelines for the management of atrial fibrillation [18]. Each subject had at least one ECG showing AF. Exclusion criteria were congenital heart disease, coronary artery disease, cardiomyopathy, concomitant valvular heart disease, previous cardiac surgery, renal insufficiency, thyroid dysfunction, acute or chronic inflammatory disease, hematological diseases, or unavailable medical records. Moreover, patients who had a recent 3-month history of blood transfusion were also excluded.

### 2.2. Study Protocol

All baseline demographic, clinical characteristics and laboratory examinations including CBC and transthoracic echocardiography were carefully recorded. CBC testing utilized clinical laboratory methods (Coulter BC-5380/6800 Hematology Analyzer, Mindray, Shenzhen, China) for white blood cell (WBC) count, red blood cell (RBC), hematocrit (HCT), mean corpuscular volume (MCV), mean corpuscular hemoglobin concentration (MCHC), platelet count (Plt), mean platelet volume (MPV), hemoglobin (Hb) levels, and RDW. Serum creatinine (Cr), uric acid (UA), and total bilirubin levels (TBIL) were measured by using an automatic blood biochemical analyzer (TBA-120 FR analyzer Toshiba, Japan). Both laboratory and biochemical tests were obtained after the second sky-abdomen examination after admission. Left atrial diameter (LAD), interventricular septal thickness (IVST), left ventricular posterior wall thickness (LVPWT), left ventricular end-diastolic diameter (LVEDD), and left ventricular ejection fraction (LVEF) were evaluated by a transthoracic echocardiographic examination (the Vivid-7 system USA).

### 2.3. Statistical Analysis

Categorical variables were reported as counts (percentage) and continuous variables as means ± standard deviation. Comparison between groups were used the Student's *t*-test or ANOVA with Tukey's post hoc test for continuous variables (as appropriate) while categorical variables were tested by Chi square. Multivariate logistic regression analysis was performed to identify the independent predictors of AF. The regression coefficients of the confounders included in the multivariable analysis were used to generate a nomogram for calculating the patient-specific probabilities of the occurrence of AF. Only *P* values < 0.05 were regarded as statistically significant. The statistical studies were carried out using Statistical Package for Social Sciences software (SPSS 24.0 for Windows, SPSS Inc., Chicago, IL, USA) and R 3.4.3 (The R Foundation, Vienna, Austria).

## 3. Results

The mean age of the included patients was 68 ± 10 years old, and 49.1% were male. There was no significant difference in age, gender, the presence of diabetes mellitus, hypertension, stroke, and smoking between the groups. Both low and high altitude, the levels of RDW and LAD in patients with AF group were higher than those in the control group (*P* < 0.05) in Tables 1 and 2, and the PeAF group was higher than PAF-group (*P* < 0.05) in Table 3. RDW, RBC, HCT, MPV, LAD, and LVEF levels were higher in the PAF group, PeAF group, or control group of high-altitude than those of corresponding groups in low altitude (Table 3).

Multivariate logistic regression analysis demonstrated that RDW, MCV, and LAD levels independently associated with AF patients in low altitude (RDW, OR 1.687, 95% CI 1.021–2.789; *P* < 0.05) in Table 4, while in high altitude, RDW, MCV, Cr, and LAD were independent predictors for AF patients (RDW, OR 1.755, 95% CI 1.179–2.613; *P* < 0.05) (Table 5).

**Figure 1:**
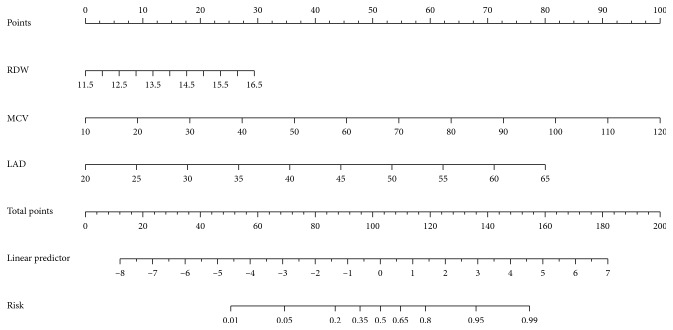
The nomogram for predicting the occurrence of AF at low altitude (3.5 m). To calculate the risk of AF, first identify the values of each axis, and than draw a vertical line upward to the point axis from each axis. Sum up the points of all variables and locate the value on to the total point line. Subsequently, draw a vertical line down to the risk of AF. AF, atrial fibrillation; RDW: red cell distribution width; MCV: mean corpuscular volume; LAD: left atrial diameter.

**Figure 2:**
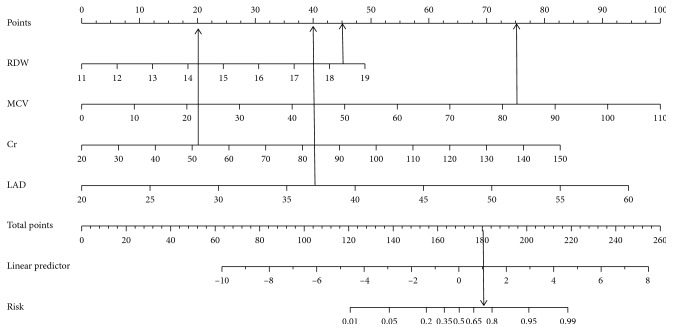
The nomogram for predicting the occurrence of AF at low altitude (2260 m). For example, the RDW (45 points), MCV (75 points), Cr (20 points), and LAD (40 points) arrive at a total of 180 points, which gives an estimated probability of 70% for occurrence of AF. AF, atrial fibrillation; RDW: red cell distribution width, MCV: mean corpuscular volume; Cr, creatinine; LAD: left atrial diameter.

Therefore, we integrated RDW, MCV, Cr, and LAD into two nomograms for prediction of probability of occurrence of AF in low altitude (Figure 1) and high altitude (Figure 2), respectively. For the nomogram, each predictor was assigned a point in the graphic interface of the nomogram, and the total points were assigned as a linear combination of the points of each predictor on a scale from 0 to 100% to find out the corresponding risk of AF.

## 4. Discussion

The main findings of our study are that AF patients have higher RDW and LAD levels than patients without AF at both high and low altitudes. Moreover, RDW and LAD levels were in high altitude compared to low altitude. Multivariate logistic regression analysis showed that RDW was a risk factor for AF.

RDW represents the variability in the size of circulating red blood cells obtained by an automatic blood count instrument, which measures 100,000 red blood cell volume over ten seconds. A number of studies have reported that RDW is a novel marker for proinflammatory reaction and oxidative stress in the body. The latter may inhibit the maturation of red blood cells, leading to the entry of immature red blood cells into the general circulation, resulting in an increase in the heterogeneity of peripheral red cell morphology [1, 9, 19]. RDW has been associated with, and used as a prognostic marker for outcomes, in many cardiovascular diseases [3–5, 20]. Liu et al. showed that not only the RDW level in patients with paroxysmal AF was significantly higher than that in the control group, but also related to the CHADS2 and CHA2DS2-VASc scores and thromboembolic risk in patients with AF [6, 21, 22]. Aksu et al. found that high RDW levels can be used to predict AF recurrence after cryoballoon ablation [23]. Moreover, previous comprehensive systematic reviews and meta-analyses confirmed that increased RDW can predict new-onset and recurrent AF generally, and after isolated coronary artery bypass grafting, valvular surgery, or combined procedures [24, 25]. This study found in two altitudes that not only RDW in patients with AF were higher than non AF patients, but also patients with persistent AF had higher levels than patients with paroxysmal AF, speculating that RDW levels were associated with AF subtypes.

The high-altitude environment results in chronic hypoxia. Several studies have shown that hypoxia causes transcription translational changes of multiple genes mediated by transcription mediators such as hypoxia-inducible factors, which in turn leads to imbalance of energy metabolism, neuroendocrine alterations, body fluid imbalance, increased oxidative stress, vascular dysfunction, and consequent pathophysiological changes [17, 26]. Indeed, exposure to acute and chronic hypoxic conditions in high altitude, increased sympathetic activation, plasma adrenaline levels, cardiac output, heart rate, and elevated blood pressure ultimately lead to cardiac structure and function change [27, 28]. In healthy people, high sympathetic activity that arises from sporting activity can induce atrial or ventricular arrhythmias through mechanisms such as increased automaticity, triggered activity, or reentry [29–31]. The commonest hematological adaptations are increased adaptive red blood cells and hemoglobin levels and polycythemia, leading to hyperviscosity, impairing the oxygen supply to multiple organs. Hypoxia can stimulate erythropoietin (EPO) synthesis and release that is the main mechanism of the occurrence of high-altitude polycythemia [26]. RDW can weaken the response of bone marrow to EPO and hinder the maturation of red blood cells, eventually leading to an increase in red blood cell volume dispersion. In our study, this may be the explanation as to why the RDW levels of each group in the high-altitude area were higher than the low altitude. The possible mechanism by which hypoxia leads to oxidative stress is increasing the generation of reactive oxygen species (ROS). Due to the limited oxygen supply, less oxygen receives electrons from oxidative phosphorylation, which may lead to accumulate reduction equivalents, and this reducing environment is beneficial to the mass production of superoxide, peroxide, and hydroxyl radicals. However, the potential source of ROS is due to the large-scale activation of xanthine oxidase and phospholipase A_2_, which not only increases the release of NO, but also increases the availability of free iron and increases the release of oxygen free radicals from red blood cells. The balance between the oxidation and antioxidant systems is broken and oxidative stress occurs when too much ROS is produced or antioxidant is depleted [17, 32–35]. Al-Hashem et al. [16] found that compared to low-altitude natural rats, serum inflammatory cytokine levels and urinary norepinephrine levels were significantly increased in high-altitude rats, and while using beta and alpha adrenergic receptor blockers, inflammatory mediator levels is lower. Hartmann et al. [36] found that at 3458 m and 4559 m, interleukin (IL) -6, IL-1, and C-reactive protein (CRP) levels were higher than baseline in healthy people in the short-term. In this study, although the WBC level in high-altitude areas is lower than that in low altitude, the level of RDW is significantly increased which is considered to be related to the sensitivity of WBC to the inflammatory state of the body is not as sensitive as high-sensitive C-reactive protein (hs-CRP) and ILs [37]. Inflammatory reaction and oxidative stress play an important role in the process of high-altitude hypoxia injury.

## 5. Limitations

Several limitations of our study should be noted. Firstly, this took a cross-sectional design without follow-up, so the impact of RDW on the progression of AF and the occurrence of AF complications has not been explored. Second, a number of inflammatory markers, such as hs-CRP, IL, and tumor necrosis factor, were not evaluated. Thirdly, the high-altitude subjects have a higher RDW level than those at low altitude, excepting hypoxia, the living habits of residents are different from those in domestic plains, which may also have an impact on RDW levels.

## 6. Conclusion

Elevated RDW levels may be an independent risk marker for nonvalvular AF, affected by type of AF and altitude. RDW is a simple and economical marker that is routinely taken during complete blood counts and could be assessed in high-altitude residents for AF risk stratification.

## Figures and Tables

**Table 1:** Baseline characteristics in all subjects at low altitude (3.5 m).

	AF (*n*=156)	Control (*n*=72)	*χ* ^2^/*T*	*P*
Male (*n* %)	77.00 (49.40)	31.00 (43.10)	0.785	0.376
Age (years)	67.92 ± 10.80	66.26 ± 10.54	0.025	0.280
Body mass index (kg/m^2^)	25.42 ± 3.93	25.26 ± 2.83	6.75	0.717
Smoking (*n* %)	22.00 (14.10)	5.00 (6.90)	2.418	0.120
Diabetes mellitus (*n* %)	31.00 (19.90)	10.00 (13.90)	1.196	0.274
Hypertension (*n* %)	97.00 (62.20)	48.00 (66.70)	0.428	0.513
Previous stroke (*n* %)	26.00 (16.70)	11.00 (15.30)	0.070	0.791
WBC (×10^9^/L)	6.86 ± 1.85	6.68 ± 1.74	1.422	0.484
RBC (×10^12^/L)	4.45 ± 0.55	4.52 ± 0.44	2.976	0.400
Hb (g/L)	139.37 ± 18.59	140.67 ± 15.98	1.008	0.610
RDW (%CV)	12.85 ± 0.78	12.59 ± 0.58	2.844	0.013
HCT (%)	41.26 ± 5.25	41.22 ± 4.48	2.26	0.954
MCV (fL)	92.81 ± 5.57	90.24 ± 9.69	0.019	0.012
MCHC (g/L)	337.67 ± 10.87	341.13 ± 8.90	2.972	0.019
Plt (×10^9^/L)	219.06 ± 58.22	222.13 ± 46.30	1.581	0.695
MPV (fL)	9.48 ± 1.00	9.33 ± 0.99	0.116	0.283
Cr (*μ*mol/L)	70.29 ± 27.62	66.29 ± 17.65	1.606	0.261
UA (mmol/L)	325.35 ± 95.49	336.63 ± 87.22	0.670	0.395
TBIL (*μ*mol/L)	14.64 ± 6.16	12.30 ± 4.18	4.203	0.001
LAD (mm)	40.45 ± 7.54	33.78 ± 4.63	14.865	0.001
LVEDD (mm)	48.34 ± 6.33	47.13 ± 5.37	1.693	0.163
IVST (mm)	9.52 ± 2.32	9.06 ± 1.87	0.461	0.148
LVPWT (mm)	9.21 ± 1.90	9.06 ± 3.09	1.503	0.636
LVEF (%)	56.70 ± 9.64	60.76 ± 5.54	7.489	0.001
Aspirin (*n* %)	105.00 (67.30)	57.00 (79.20)	3.368	0.066
ACEIs or ARBs (*n* %)	49.00 (31.40)	19.00 (26.40)	0.593	0.441
*β*-blocker (*n* %)	67.00 (42.90)	28.00 (38.90)	0.334	0.563
CCBs (*n* %)	51.00 (32.70)	22.00 (30.60)	0.103	0.748
Statins (*n* %)	71.00 (45.50)	38.00 (52.80)	1.042	0.307

AF, atrial fibrillation; BMI, body mass index; WBC, white blood cell; RBC, red blood cell; Hb, hemoglobin; RDW, red cell distribution width; HCT, hematocrit; MCV, mean corpuscular volume; MCHC, mean corpuscular hemoglobin concentration; Plt, platelet count; MPV, mean platelet volume; Cr, creatinine; UA, uric acid; TBIL, total bilirubin; LAD, left atrial diameter; LVEDD, left ventricular end-diastolic diameter; IVST, interventricular septal thickness; LVPWT, left ventricular posterior wall thickness; LVEF, left ventricular ejection fraction.

**Table 2:** Baseline characteristics in all subjects at high altitude (2260 m).

	AF (*n*=147)	Control (*n*=95)	*χ* ^2^/*T*	*P*
Male (*n* %)	85.00 (57.80)	46.00 (48.40)	2.055	0.152
Age (years)	68.16 ± 9.58	69.28 ± 8.99	1.359	0.363
Body mass index (kg/m^2)^	25.55 ± 3.43	25.97 ± 2.64	4.467	0.290
Smoking (*n* %)	25.00 (17.00)	11.00 (11.60)	1.343	0.247
Diabetes mellitus (*n* %)	30.00 (20.40)	14.00 (14.70)	1.248	0.264
Hypertension (*n* %)	104.00 (70.70)	64.00 (67.40)	0.311	0.577
Previous stroke (*n* %)	32.00 (21.80)	15.00 (15.80)	1.318	0.251
WBC (×10^9^/L)	5.63 ± 1.56	5.72 ± 1.63	0.004	0.668
RBC (×10^12^/L)	4.91 ± 0.68	4.92 ± 0.57	2.798	0.824
Hb (g/L)	152.66 ± 19.07	150.98 ± 17.28	1.245	0.488
RDW (% CV)	13.83 ± 1.12	13.10 ± 0.85	5.765	0.001
HCT (%)	46.26 ± 5.64	45.82 ± 5.01	1.726	0.538
MCV (fL)	94.56 ± 4.93	92.52 ± 9.75	0.110	0.032
MCHC (g/L)	330.24 ± 9.84	330.21 ± 9.44	0.038	0.983
Plt (×10^9^/L)	154.60 ± 52.49	180.98 ± 47.62	0.342	0.001
MPV (fL)	12.16 ± 1.52	11.96 ± 1.26	0.041	0.302
Cr (*μ*mol/L)	80.15 ± 20.68	63.47 ± 14.12	10.889	0.001
UA (mmol/L)	374.16 ± 114.71	331.38 ± 83.48	7.007	0.001
TBIL (*μ*mol/L)	18.00 ± 8.00	16.93 ± 8.09	0.115	0.316
LAD (mm)	41.58 ± 6.56	34.21 ± 4.14	15.665	0.001
LVEDD (mm)	46.23 ± 4.73	46.01 ± 3.78	4.54	0.689
IVST (mm)	10.44 ± 1.49	9.88 ± 1.30	3.288	0.004
LVPWT (mm)	10.18 ± 1.30	9.66 ± 1.17	0.306	0.002
LVEF (%)	64.70 ± 6.89	64.57 ± 5.70	3.524	0.876
Aspirin (*n* %)	107.00 (72.80)	59.00 (62.10)	3.058	0.080
ACEIs or ARBs (*n* %)	40.00 (27.20)	16.00 (16.80)	3.488	0.062
*β*-blocker (*n* %)	55.00 (37.40)	25.00 (26.30)	3.212	0.073
CCBs (*n* %)	49.00 (33.30)	30.00 (31.60)	0.081	0.776
Statins (*n* %)	65.00 (44.20)	39.00 (41.10)	0.236	0.627

AF, atrial fibrillation; BMI, body mass index; WBC, white blood cell; RBC, red blood cell; Hb, hemoglobin; RDW, red cell distribution width; HCT, hematocrit; MCV, mean corpuscular volume; MCHC, mean corpuscular hemoglobin concentration; Plt, platelet count; MPV, mean platelet volume; Cr, creatinine; UA, uric acid; TBIL, total bilirubin; LAD, left atrial diameter; LVEDD, left ventricular end-diastolic diameter; IVST, interventricular septal thickness; LVPWT, left ventricular posterior wall thickness; LVEF, left ventricular ejection fraction.

**Table 3:** Baseline characteristics in patients with AF at two altitudes.

	Low altitude (3.5 m)			High altitude (2260 m)			*P*
	PAF (*n*=77)	PeAF (*n*=79)	Control (*n*=72)	PAF (*n*=77)	PeAF (*n*=70)	Control (*n*=95)	
Male (*n* %)	32.00 (41.60)	45.00 (57.00)	31.00 (43.10)	48.00 (62.30)	37.00 (52.90)	46.00 (48.40)	0.075
Age (years)	66.92 ± 12.61	68.89 ± 8.66	66.26 ± 10.54	67.23 ± 10.67	69.19 ± 8.17	69.28 ± 8.99	0.250
BMI (kg/m^2)^	25.10 ± 4.02	25.74 ± 3.84	25.26 ± 2.83	25.18 ± 3.63	25.96 ± 3.18	25.97 ± 2.64	0.351
Smoking (*n* %)	11.00 (14.30)	11.00 (13.90)	5.00 (6.90)	12.00 (15.60)	13.00 (18.60)	11.00 (11.60)	0.434
Diabetes mellitus (*n* %)	11.00 (14.30)	20.00 (25.30)	10.00 (13.90)	16.00 (20.80)	14.00 (20.00)	14.00 (14.70)	0.340
Hypertension (*n* %)	46.00 (59.70)	51.00 (64.60)	48.00 (66.70)	55.00 (71.40)	49.00 (70.00)	64.00 (67.40)	0.704
Previous stroke (*n* %)	10.00 (13.00)	16.00 (20.30)	11.00 (15.30)	13.00 (16.90)	19.00 (27.10)	15.00 (15.80)	0.276
WBC (×10^9^/L)	6.89 ± 1.92	6.82 ± 1.79	6.68 ± 1.74	5.56 ± 1.78^a^	5.70 ± 1.28^a^	5.72 ± 1.63^a^	0.001
RBC (×10^12^/L)	4.40 ± 0.53	4.51 ± 0.58	4.52 ± 0.44	4.90 ± 0.75^a^	4.92 ± 0.59^a^	4.92 ± 0.57^a^	0.001
Hb (g/L)	138.48 ± 17.28	140.24 ± 19.85	140.67 ± 15.98	152.55 ± 19.45^a^	152.79 ± 18.78^a^	150.98 ± 17.28^a^	0.001
RDW (%CV)	12.71 ± 0.74	12.98 ± 0.80^c^	12.59 ± 0.58	13.52 ± 0.99^a^	14.17 ± 1.16^abc^	13.10 ± 0.85^a^	0.001
HCT (%)	40.55 ± 4.75	41.97 ± 5.64	41.22 ± 4.47	46.13 ± 5.68^a^	46.40 ± 5.64^a^	45.82 ± 5.01^a^	0.001
MCV (fL)	92.37 ± 5.12	93.24 ± 5.98^c^	90.24 ± 9.69	94.69 ± 5.26^ac^	94.42 ± 4.57	92.52 ± 9.75^a^	0.002
MCHC (g/L)	341.39 ± 9.97	334.04 ± 10.52	341.13 ± 8.90	330.94 ± 10.05^a^	329.47 ± 9.62^a^	330.21 ± 9.44^a^	0.001
Plt (×10^9^/L)	227.84 ± 62.69	210.51 ± 52.50^b^	222.13 ± 46.30	156.94 ± 49.28^ac^	152.03 ± 56.06^ac^	180.98 ± 47.62^a^	0.001
MPV (fL)	9.37 ± 0.97	9.58 ± 1.02	9.33 ± 0.99	12.04 ± 1.44^a^	12.29 ± 1.60^a^	11.96 ± 1.26^a^	0.001
Cr (*μ*mol/L)	69.73 ± 32.44	70.83 ± 22.15	66.29 ± 17.65	81.05 ± 22.14^ac^	79.16 ± 19.05^ac^	63.47 ± 14.12	0.001
UA (mmol/L)	310.62 ± 94.97	339.70 ± 94.39	336.63 ± 87.22	362.09 ± 100.77^a^	387.44 ± 127.74^c^	331.38 ± 83.48	0.001
TBIL (*μ*mol/L)	13.47 ± 6.07	15.79 ± 0.06^c^	12.30 ± 4.18	16.43 ± 5.87^a^	19.73 ± 9.57	16.93 ± 8.09	0.001
LAD (mm)	37.00 ± 6.48^c^	43.82 ± 6.99^bc^	33.78 ± 4.63	38.08 ± 4.95^c^	45.43 ± 5.95^bc^	34.21 ± 4.14	0.001
LVEDD (mm)	46.83 ± 5.34	49.81 ± 6.88^b^	47.13 ± 5.38	45.97 ± 4.11	46.51 ± 5.34^a^	46.01 ± 3.80	0.001
IVST (mm)	9.45 ± 2.25	9.58 ± 2.41	9.06 ± 1.87	10.48 ± 1.60^a^	10.39 ± 1.37	9.88 ± 1.30^a^	0.001
LVPWT (mm)	9.26 ± 2.18	9.17 ± 1.60	9.06 ± 3.09	10.21 ± 1.40^a^	10.14 ± 1.20^a^	9.66 ± 1.17	0.001
LVEF (%)	59.47 ± 6.63	54.00 ± 11.27	60.76 ± 5.54	65.68 ± 7.10^a^	63.63 ± 6.52^a^	64.57 ± 5.70^a^	0.001
Aspirin (*n* %)	51.00 (62.20)	54.00 (68.40)	57.00 (79.20)	55.00 (71.40)	52.00 (74.30)	59.00 (62.10)	0.223
ACEIs or ARBs (*n* %)	22.00 (28.60)	27.00 (34.20)	19.00 (26.40)	19.00 (24.70)	21.00 (30.00)	16.00 (16.80)	0.174
*β*-blocker (*n* %)	35.00 (45.50)	32.00 (40.50)	28.00 (38.90)	28.00 (36.40)	27.00 (38.60)	25.00 (26.30)	0.180
CCBs (*n* %)	24.00 (31.20)	27.00 (34.20)	22.00 (30.60)	26.00 (33.80)	23.00 (32.90)	30.00 (31.60)	0.996
Statins (*n* %)	41.00 (53.20)	30.00 (38.00)	38.00 (52.80)	30.00 (39.00)	35.00 (50.00)	30.00 (31.60)	0.017

AF, atrial fibrillation; PAF, paroxysmal AF; PeAF, persistent AF; BMI, body mass index; WBC, white blood cell; RBC, red blood cell; Hb, hemoglobin; RDW, red cell distribution width; HCT, hematocrit; MCV, mean corpuscular volume; MCHC, mean corpuscular hemoglobin concentration; Plt, platelet count; MPV, mean platelet volume; Cr, creatinine; UA, uric acid; TBIL, total bilirubin; LAD, left atrial diameter; LVEDD, left ventricular end-diastolic diameter; IVST, interventricular septal thickness; LVPWT, left ventricular posterior wall thickness; LVEF, left ventricular ejection fraction; a: compared to the different altitude correspondence groups; b: compared to the same altitude PAF-group; c: compared to the same altitude control group.

**Table 4:** Multiple logistic regression analysis to detect the independent predictors of the occurrence of AF at low altitude (3.5 m).

Variables	*β*	Wald *χ*^2^	*P*	OR	95% CI
RDW	0.523	4.160	0.041	1.687	1.021∼2.789
MCV	0.074	3.956	0.047	1.077	1.001∼1.159
TBIL	0.031	0.774	0.379	1.031	0.963∼1.104
LAD	0.142	21.823	0.001	1.152	1.086∼1.223
LVEF	−0.032	1.718	0.190	0.968	0.923∼1.016

RDW, red cell distribution width; MCV, mean corpuscular volume; TBIL, total bilirubin; LAD, left atrial diameter; LVEF, left ventricular ejection fraction.

**Table 5:** Multiple logistic regression analysis to detect the independent predictors of the occurrence of AF at high altitude (2260 m).

Variables	*β*	Wald *χ*^2^	*P*	OR	95% CI
RDW	0.562	7.669	0.006	1.755	1.179∼2.613
MCV	0.084	4.251	0.039	1.088	1.004∼1.178
Hb	0.003	0.091	0.762	1.003	0.975∼1.019
Cr	0.063	20.331	0.001	1.065	1.036∼1.094
UA	0.001	0.070	0.791	1.001	0.996∼1.003
LAD	0.233	30.785	0.001	1.262	1.162∼1.370
IVST	0.133	0.163	0.687	1.142	0.599∼2.180
LVPWT	0.218	0.330	0.565	1.244	0.382∼1.692

RDW, red cell distribution width; MCV, mean corpuscular volume; Hb, hemoglobin; Cr, creatinine; UA, uric acid; LAD, left atrial diameter; IVST, interventricular septal thickness; LVPWT, left ventricular posterior wall thickness.

## Data Availability

The data of this study can be obtained from the corresponding author.
